# Improved Tau PET SUVR Quantification in 4-Repeat Tau Phenotypes with [^18^F]PI-2620

**DOI:** 10.2967/jnumed.123.265930

**Published:** 2024-06

**Authors:** Gérard N. Bischof, Matthias Brendel, Henryk Barthel, Hendrik Theis, Michael Barbe, Peter Bartenstein, Joseph Claasen, Adrian Danek, Günter Höglinger, Johannes Levin, Ken Marek, Bernd Neumaier, Carla Palleis, Marianne Patt, Michael Rullmann, Dorothee Saur, Matthias L. Schroeter, John Seibyl, Mengmeng Song, Andrew Stephens, Osama Sabri, Alexander Drzezga, Thilo van Eimeren, for the German Imaging Initiative for Tauopathies

**Affiliations:** 1 Department of Nuclear Medicine, University Hospital Cologne, Cologne, Germany;; 2 Molecular Organization of the Brain, Institute for Neuroscience and Medicine, Jülich, Germany;; 3 German Center for Neurodegenerative Diseases, Munich, Germany;; 4 Munich Cluster for Systems Neurology, Munich, Germany;; 5 Department of Nuclear Medicine, University Hospital of Munich, LMU Munich, Munich, Germany;; 6 Department of Nuclear Medicine, University Hospital of Leipzig, Leipzig, Germany;; 7 Department of Neurology, Faculty of Medicine and University Hospital Cologne, University of Cologne, Cologne, Germany;; 8 Department of Neurology, University Hospital of Munich, LMU Munich, Munich, Germany;; 9 InviCRO, LLC, Boston, Massachusetts;; 10 Molecular Neuroimaging, a division of inviCRO, New Haven, Connecticut;; 11 Institute of Radiochemistry and Experimental Molecular Imaging, University of Cologne, Cologne, Germany;; 12 Institute of Neuroscience and Medicine, Nuclear Chemistry, Research Center Jülich, Jülich, Germany;; 13 Clinic for Cognitive Neurology, University Hospital of Leipzig, and Max Planck Institute for Human Cognitive and Brain Sciences, Leipzig, Germany;; 14 Life Molecular Imaging GmbH, Berlin, Germany; and; 15 German Center for Neurodegenerative Diseases, Bonn/Cologne, Germany

**Keywords:** molecular imaging, neurology, PET, 4R, clinical severity, tau PET

## Abstract

We used a new data-driven methodology to identify a set of reference regions that enhanced the quantification of the SUV ratio of the second-generation tau tracer 2-(2-([^18^F]fluoro)pyridin-4-yl)-9H-pyrrolo[2,3-b:4,5-c′]dipyridine ([^18^F]PI-2620) in a group of patients clinically diagnosed with 4-repeat tauopathy, specifically progressive supranuclear palsy or cortical basal syndrome. The study found that SUV ratios calculated using the identified reference regions (i.e., fusiform gyrus and crus-cerebellum) were significantly associated with symptom severity and disease duration. This establishes, for the first time to our knowledge, the suitability of [^18^F]PI-2620 for tracking disease progression in this 4-repeat disease population. This is an important step toward increased clinical utility, such as patient stratification and monitoring in disease-modifying treatment trials. Additionally, the applied methodology successfully optimized reference regions for automated detection of brain imaging tracers. This approach may also hold value for other brain imaging tracers.

## Visual Abstract

**Figure:**
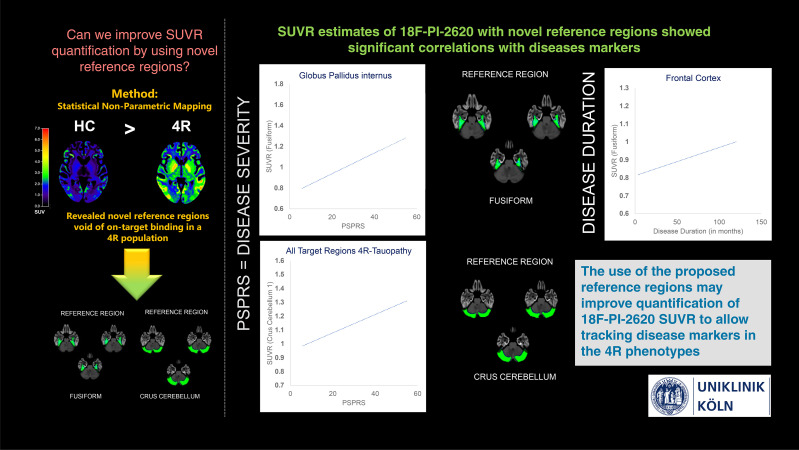

PET imaging with 2-(2-([^18^F]fluoro)pyridin-4-yl)-9H-pyrrolo[2,3-b:4,5-c′]dipyridine ([^18^F]PI-2620) has demonstrated the ability to detect tau pathology in patients diagnosed with progressive supranuclear palsy (PSP) (1) and corticobasal syndrome (2,3). This holds potential for refining diagnostic criteria for 4-repeat (4R) tau isoforms and addressing diagnostic challenges associated with overlapping symptoms in neurodegenerative diseases such as frontotemporal dementia and Parkinson disease (4). Although [^18^F]PI-2620 meets certain criteria for an ideal biomarker, such as positivity in the symptomatic phase and specificity for pathology variants, its binding affinity and relationship with 4R tau pathology have shown some discrepancies (4,5).

Previous attempts to establish a correlation between [^18^F]PI-2620 binding potentials and disease progression or severity have been inconclusive. A potential source of variance in SUV ratio (SUVR) sensitivity is the choice of reference region. The use of the cerebellar cortex as a reference region, common in Alzheimer disease studies, is less ideal in 4R phenotypes because of known on-target binding in the dentate nucleus (6,7). To address this issue, we used a data-driven approach akin to established count normalization procedures. This involved identifying regions in PSP patients devoid of pathology for effective count normalization (8). Criteria for region suitability included absence of on-target binding, plausibility (e.g., bilateral), lack of on-target binding in early disease histopathologic studies, and correlation of SUVRs with disease severity or duration. This approach aims to enhance the sensitivity of SUVR measurements by selecting reference regions that better represent areas free of pathology in PSP patients. By refining the reference region choice, we anticipate improved accuracy in assessing the relationship between [^18^F]PI-2620 binding potentials and disease measures. This strategy contributes to the ongoing efforts to establish reliable biomarkers for neurodegenerative diseases, particularly those involving 4R tau isoforms.

## MATERIALS AND METHODS

In this study, 43 patients with suspected 4R tauopathies (PSP or corticobasal syndrome) and 14 healthy controls were enrolled from 4 nuclear medicine clinics in Germany and the United States. Diagnosis for PSP and corticobasal syndrome followed established criteria (9,10). All participants underwent a T1-weighted 3-dimensional MRI sequence (1 × 1 × 1 mm; 256 slices) and dynamic PET imaging (0–90 min) of [^18^F]PI-2620 PSP severity (PSP rating scale), and disease duration data were available for most patients. Ethical approval was obtained from institutional committees, and participants provided written informed consent before the PET scans.

**FIGURE 1.**
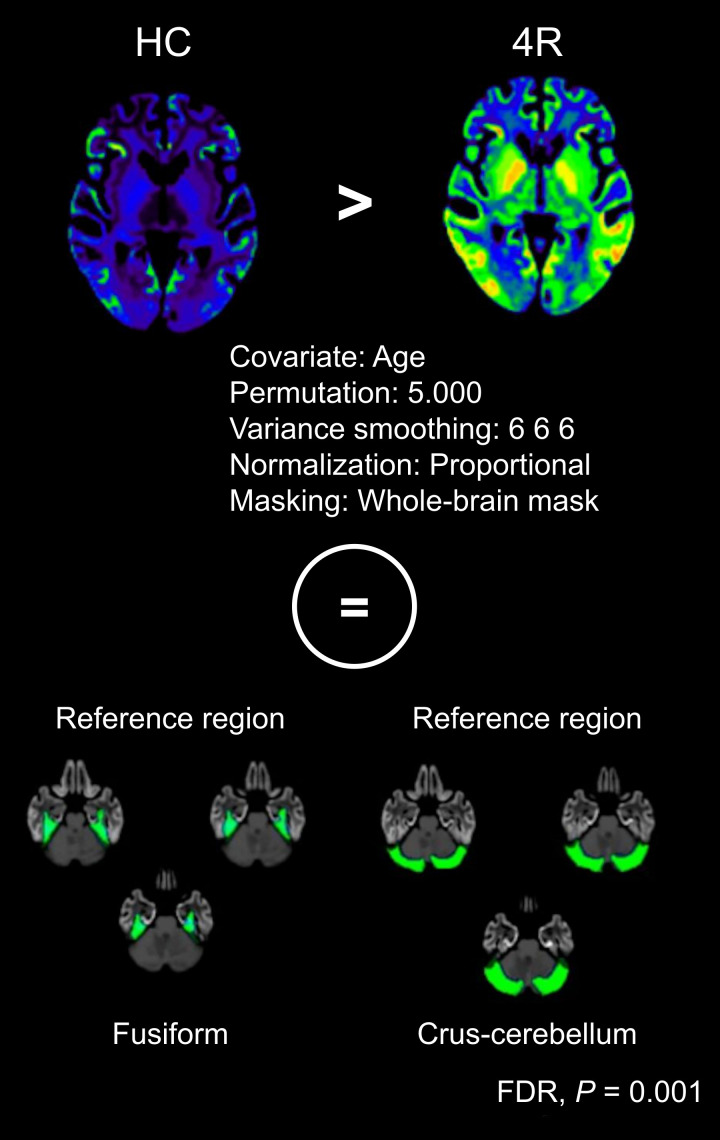
Methodologic approach of SnPM comparing healthy controls (HC) vs. patients with suspected 4R tauopathy. This statistical approach allowed us to identify reference regions void of on-target binding in patient population. Interestingly, we identified fusiform gyrus and crus-cerebellum to show nonspecific binding with using false-discovery rate of *P* = 0.01.

Participants in the healthy control cohort were amyloid-negative (based on cerebrospinal fluid or PET information) and were cognitively normal (as assessed by the Mini–Mental State Examination). Further details on the cohort have been published previously (3,4). Dynamic PET images (0–60 min) of [^18^F]PI-2620 were realigned, the 30- to 60-min frames were averaged, and the resulting SUV images were coregistered to the individual MR images. PET images were normalized and partial-volume–corrected using the geometric transfer method (11). Partial-volume–corrected PET images were submitted to the statistical nonparametric mapping toolbox (SnPM (12)) implemented in SPM to identify regions in the patient population clearly void of pathology and therefore most effective for count normalization. SnPM was chosen because of the relatively lower degrees of freedom in the healthy control cohort and to capitalize on the individual patient data and the strong data-driven nature of our approach. Nonparametric permutation was set to 5,000 iterations, with a variance smoothing of 6 mm^3^. Age served as a covariable, and a whole-brain inclusion mask was applied. The false-discovery rate was set at a *P* value of 0.01 (Fig. 1), and resulting clusters were regionally labeled on the basis of the automated anatomic atlas. In the patient cohort, automated anatomic atlas regions were used as reference regions, and SUVRs were extracted from target regions shown to accumulate tau pathology in 4R phenotypes (1,3). To evaluate the sensitivity of the data-driven reference regions, we performed partial correlation analyses of SUVRs in target regions, with the PSP rating scale and disease duration corrected for age (*R*_(age)_).

## RESULTS

**FIGURE 2.**
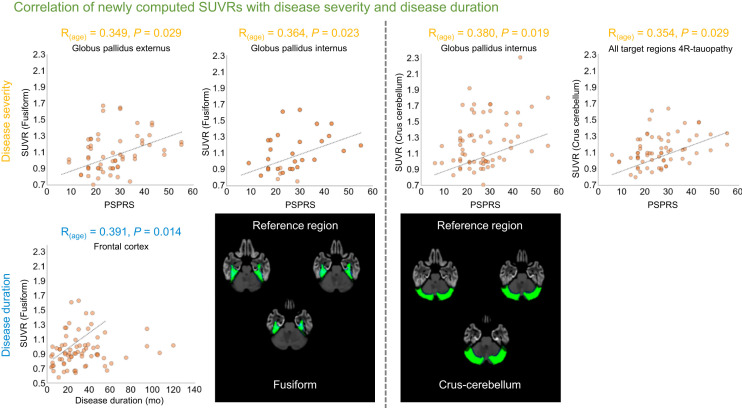
Significant partial correlations of newly computed SUVRs, using fusiform or crus-cerebellum as reference region, with disease severity (PSP rating scale [PSPRS]; upper panels) or disease duration (in months; lower panels). Automated anatomic atlas reference regions are overlayed on standard MRI template.

**FIGURE 3.**
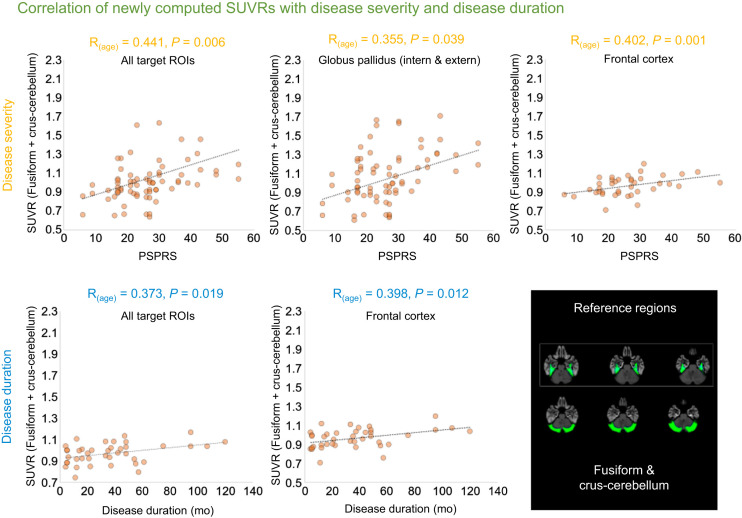
Significant partial correlations of combined reference regions (fusiform or crus-cerebellum as reference region), with disease severity (PSP rating scale [PSPRS]; upper panels) or disease duration (in months; lower panels). Automated anatomic atlas reference regions are overlayed on standard MRI template.

Demographic characteristics of the 43 patients and 14 healthy controls are summarized in Table 1. The statistical nonparametric mapping approach revealed differences, with elevated nonspecific binding bilaterally in the fusiform gyrus and crus-cerebellum, when comparing healthy controls versus 4R patients (Fig. 1). These regions were used as reference regions (separately and in combination), and SUVRs were computed. The partial correlation analysis (Figs. 2 and 3) using SUVRs based on the fusiform gyrus reference region revealed a significant association between disease severity and accumulating tau pathology in the globus pallidus externus (*R*_(age)_ = 0.34, *P* = 0.02) and internus (*R*_(age)_ = 0.36, *P* = 0.02). Disease duration was significantly associated with tau pathology in the frontal cortex (*R*_(age)_ = 0.39, *P* = 0.01). For the crus-cerebellum, SUVR estimates in the globus pallidus internus (*R*_(age)_ = 0.38, *P* = 0.01) and averaged across all target regions (*R*_(age)_ = 0.35, *P* = 0.02) showed significant associations with disease severity, whereas no significant correlations with disease duration were observed. The combination of both reference regions (Fig. 3) revealed several significant correlation coefficients with both disease severity (*R*_(age)_ for all target ROIs = 0.44, *P* = 0.01; *R*_(age)_ for frontal cortex = 0.40, *P* = 0.01; *R*_(age)_ for globus pallidus externus = 0.35, *P* = 0.03; *R*_(age)_ for globus pallidus internus = 0.37, *P* = 0.02; *R*_(age)_ for globus pallidus = 0.36, *P* = 0.03) and disease duration (*R*_(age)_ for all target ROIs = 0.37, *P* = 0.02; *R*_(age)_ for frontal cortex = 0.40, *P* = 0.01).

**TABLE 1.** Patient and Control Demographics

			4R phenotype	
Demographic	Control	4R	PSP	Corticobasal syndrome
*n*	14	43	31	12
Age (y)	64.38 (10.36)	70.6 (7.9)	70.8 (7.7)	69.6 (8.4)
Mini–Mental State Examination	29.07 (1.14)	NA	NA	NA
Montreal cognitive assessment	NA	22.3 (5.0)	22.9 (4.4)	20.4 (6.1)
PSP rating scale	NA	26.03 (10.0)	27.1 (10.5)	22.7 (7.6)
Disease duration (mo)	NA	36.48 (29.2)	37.1 (32.1)	34.5 (17.9)

NA= not applicable.

Data are mean followed by SD in parentheses.

## DISCUSSION

Here we show that using a data-driven statistical nonparametric approach to isolate regions that could serve as a potential reference region revealed several findings: regions with no on-target binding in the cohort of 4R phenotypes, plausibility due to the bilateral voxels located in both the fusiform gyrus and the crus-cerebellum, and no on-target binding based on histopathologic studies. In addition, SUVRs in target regions using either reference region showed significant relationships with clinical measures. The globus pallidus internus/externus was previously identified as the region best suited to discriminate different tauopathies using [^18^F]PI-2620 (1–3), whereas our study expanded on these findings by showing sensitivity of the pallidum to disease severity measures. Additionally, the correlation with frontal tau pathology and disease duration adds plausibility to our reference regions, as cortical involvement of 4R tauopathy is known to be an indication of advanced disease stage (6). We therefore propose the fusiform gyrus and the crus-cerebellum as 2 potential candidates for further exploration as potential reference regions for [^18^F]PI-2620 in a larger independent sample of 4R patients and in individuals for whom arterial blood sampling is available to further evaluate the suitability of our reference regions in 4R tauopathies.

We provide evidence that the previously established normalization count method (8) within the statistical nonparametric mapping context may be a sensitive automated approach to probe potential candidates for reference regions, particularly if the sample size of the target population is limited and the distribution of the tracer retention is relatively heterogeneous. Our results are important in the context of clinical trials of potential disease-modifying therapies in 4R tauopathies (13), as treatment effects could be quantified both on the pathophysiologic scale using [^18^F]PI-2620 and according to disease severity. Our use of partial-volume–corrected data in our analysis may be a limitation because of the accessibility of high-resolution magnetization-prepared, rapid gradient-echo imaging in the clinical context.

## CONCLUSION

Whereas recent studies have shown that the regional uptake pattern of [^18^F]PI-2620 can assist in the differential diagnosis of primary versus secondary tauopathies, here we extend the utility of [^18^F]PI-2620. Specifically, we show that different reference regions (i.e., fusiform gyrus, crus-cerebellum, and the combination) may improve SUVR quantification, as our newly computed SUVRs in target regions of 4R tauopathies relate to both disease severity and disease duration. We recognize that in the absence of specific criteria for appropriate reference regions, the criteria we have chosen may not be exhaustive for determining an appropriate reference region, but they may provide some initial guidance.

## Notes

KEY POINTS **QUESTION:** Can we improve SUVR sensitivity in 4R phenotypes using data-driven reference regions? **PERTINENT FINDINGS:** We found suitable reference regions (i.e., crus-cerebellum and fusiform gyrus) in 4R phenotypes that led to SUVR estimates in target regions of 4R tauopathy that tracked with disease severity and duration. **IMPLICATIONS FOR PATIENT CARE:** Our results improve the utility of ^18^F-PI-2620 to quantify tau burden, which will improve the clinical application of ^18^F-PI-2620 in the differential diagnosis of tauopathies.
